# Commensal *Streptococcus salivarius* Modulates PPARγ Transcriptional Activity in Human Intestinal Epithelial Cells

**DOI:** 10.1371/journal.pone.0125371

**Published:** 2015-05-06

**Authors:** Benoît Couvigny, Tomas de Wouters, Ghalia Kaci, Elsa Jacouton, Christine Delorme, Joël Doré, Pierre Renault, Hervé M. Blottière, Eric Guédon, Nicolas Lapaque

**Affiliations:** 1 INRA, UMR 1319 MICALIS, Domaine de Vilvert, Jouy-en-Josas, France; 2 AgroParisTech, UMR Micalis, Jouy-en-Josas, France; 3 INRA, US 1367 MetaGenoPolis, Jouy-en-Josas, France; ContraFect Corporation, UNITED STATES

## Abstract

The impact of commensal bacteria in eukaryotic transcriptional regulation has increasingly been demonstrated over the last decades. A multitude of studies have shown direct effects of commensal bacteria from local transcriptional activity to systemic impact. The commensal bacterium *Streptococcus salivarius* is one of the early bacteria colonizing the oral and gut mucosal surfaces. It has been shown to down-regulate nuclear transcription factor (NF-кB) in human intestinal cells, a central regulator of the host mucosal immune system response to the microbiota. In order to evaluate its impact on a further important transcription factor shown to link metabolism and inflammation in the intestine, namely PPARγ (peroxisome proliferator-activated receptor), we used human intestinal epithelial cell-lines engineered to monitor PPARγ transcriptional activity in response to a wide range of *S*. *salivarius* strains. We demonstrated that different strains from this bacterial group share the property to inhibit PPARγ activation independently of the ligand used. First attempts to identify the nature of the active compounds showed that it is a low-molecular-weight, DNase-, proteases- and heat-resistant metabolite secreted by *S*. *salivarius* strains. Among PPARγ-targeted metabolic genes, *I-FABP* and *Angptl4* expression levels were dramatically reduced in intestinal epithelial cells exposed to *S*. *salivarius* supernatant. Both gene products modulate lipid accumulation in cells and down-regulating their expression might consequently affect host health. Our study shows that species belonging to the salivarius group of streptococci impact both host inflammatory and metabolic regulation suggesting a possible role in the host homeostasis and health.

## Introduction

The Human gastrointestinal tract (GIT) harbors a highly diverse and dense population of commensal microorganisms, commonly named microbiota. Its functions were formerly thought to be purely digestive and protective by building a competitive barrier against pathogen colonization. Research performed over the last decades has given rise to an emergent awareness that the function of the GIT along with its microbiota also strongly influences host physiology, locally and at a systemic level contributing largely to the host health and wellbeing (for review see [1]). The interface between commensal bacteria and the host epithelium is crucial for the establishment of this interaction in a homeostatic and mutualistic manner. With a large genetic pool (over 150 time larger than the Human genome), the microbiota is highly adapted for intestinal fermentation of non-digestible foodstuff [2]. On top of this important fermentative role it contributes to the development of the local and systemic immune system, to the regulation of host fat storage and even to behavior [3–9]. Strong correlations between the microbiota, low-grade inflammation and host metabolism have been highlighted recently [4, 10, 11]. However, the understanding of underlying mechanisms by which the gut microbiota could contribute to the host metabolic homeostasis or functions remains fragmentary.

An important role has been attributed to metabolites produced by the microbiota (including short chain fatty acids (SCFA)) in the activation of peroxisome proliferator-activated receptor (PPAR) family of nuclear receptors that initiate transcriptional gene expression linked to metabolic reprogramming and immune functions [12–15]. PPARγ is a well-characterized nuclear receptor for which natural known ligands are endogenous and exogenous lipid moieties along with derivatives of thiazolidinedione [16]. PPARγ forms heterodimers with the retinoid X receptor (RXR) and upon activation stimulates target-gene expression through binding to PPAR-responsive elements (PPREs) [17]. PPARγ is predominantly expressed in adipose tissue and the GIT, and is involved in the metabolic regulation of lipids, glucose homeostasis, cell proliferation and differentiation and local inflammation. Microbiota-induced PPARγ has also a role beyond the gut, as it regulates *Angptl4* (Angiopoietin like protein-4) expression, responsible for lipid storage in the adipose tissues [12, 18]. In addition to its implication in metabolic gene regulation, PPARγ is a well-characterized transcription factor recently reported to act as an E3 ubiquitin ligase implicated in the degradation of the p65 sub-unit of the pleiotropic nuclear factor NF-κB that consequently reduces inflammatory responses [19, 20].

*Streptococcus salivarius* is one the primo colonizers of the human oral cavity and upper airways where it remains a predominant commensal inhabitant [21–23]. This bacterium is also a dominant part of the early-life human intestinal microbiota (for review see [24]). In adults, *S*. *salivarius* colonizes the stomach and intestine mucosal surfaces including the ileum, jejunum and colon [23, 25–28]. Metagenomic and phylogenetic microarray approaches showed that *Streptococcus* species are ubiquitously present in ileum and colon of healthy adults in dominance and sub-dominance respectively [29–32]. Previously, our laboratory demonstrated that *S*. *salivarius* strains display regulatory effects on the NF-κB pathway in human intestinal epithelial cells via unknown pathways and protect its host during induced-colitis suggesting a potential role in GIT inflammatory homeostasis [33, 34]. Interestingly, pathogen and commensal bacteria have been shown to actively modulate PPARγ and NF-κB activation [13, 20, 35–38]. Altogether, these studies suggest that, NF-κB and PPARγ are transcription factors, influenced by bacteria, with distinct but overlapping roles linking metabolic and immune functions. These observations prompted us to investigate the effect of *S*. *salivarius* on PPARγ transcriptional activity. In the present study, we showed that *S*. *salivarius* strains down-regulate PPARγ transcriptional activity and PPARγ-dependent genes expression *via* low molecular weight, heat resistant, non peptidic and non nucleotidic secreted components.

## Materials and Methods

### Epithelial cell culture

The human epithelial cell lines HT-29, Caco-2 and SW-116 were obtained from the American Type Culture Collection (ATCC). HT-29 and SW-116 were cultured in RPMI 1640 and Caco-2 in DMEM, supplemented with 10% heat-inactivated foetal calf serum, 2mM glutamine, 1X non essential amino acids, penicillin (50U/ml) and streptomycin (50U/ml) in a humidified 5% CO2 atmosphere at 37°C. All culture media and supplements were supplied by Lonza.

### Reagents

All PPARγ ligands and antagonist: Rosiglitazone (10μM), GW9662 (10μM) pioglitazone (10μM), troglitazone (10μM) and ciglitazone (10μM) (Cayman chemical) were prepared in DMSO following the manufacturer’s recommendations and diluted in RPMI. Sodium butyrate (But, 2mM) was from Sigma-aldrich.

### Bacterial culture and bacterial supernatant preparation

The *Streptococcus salivarius* strains used in this study are listed in S1 Table. Bacteria were grown overnight at 37°C, *S*. *salivarius*, *S*. *vestibularis* and *S*. *agalactiae* in chemically defined medium (CDM) [39] and *E*. *coli* in Luriani Broth (LB). Bacterial cultures supernatants were collected after centrifugation and 0.22μm filtration. CDM adjusted to bacterial supernatant pH (pH≈5.5) was used as control. When mentioned, bacterial supernatants were size-fractionated using 10kDa and 3kDa cut-off filters (Millipore) or exposed to high temperature (100°C for 10 min) and heat shock assay (100°C/10 min prior to liquid nitrogen-freezing). Bacterial lysates were prepared by mechanical lysis using a FastPrep instrument (MP Biomedicals). 3kDa filtered bacterial supernatants and CDM were treated with proteinase K (100μg/ml, Sigma), DNase I (100μg/ml, Sigma) or trypsin (0,25%) for 2h at 37°C. Then, based on their molecular weight characteristics ranging from 23 to 29 kDa, the enzymes were eliminated by using a 10kDa cut-off filters.

### Luciferase Reporter and cell viability Assays

PPARγ reporter construct pJ3-TK-Luc bearing three repeats of the PPRE (PPARγ responsive element) [40] was used to establishing HT-29-PPARγ and Caco-2-PPARγ reporter cell lines as described [41]. PPARγ reporter system response was characterised using a wide range of known agonists: rosiglitazone, pioglitazone, troglitazone and ciglitazone and its well-characterised antagonist GW9662 (S3 Fig). ANGPTL4-reporter system containing the pANGPTL4-1.8luc was previously described [12]. Cell reporters were seeded in 96-well plates at 3.10^4^ cells per well. After 24h culture, bacterial supernatants or lysates, CDM, PPARγ agonists/antagonists were added at 10% final volume for 12h. For the live bacteria experiments, *S*. *salivarius* over-night cultures were incubated at a MOI of 40 bacteria per cell for 6h. Luciferase activity was measured in cells lysates using Tecan Infinite M200 device and luciferase assay kit (One Glo, Promega) according to the manufacturer’s instructions. PPARγ activation and *Angptl4* expression were normalized to controls, i.e. cells stimulated with activators in addition to CDM control media or CDM control media alone. Experiments were performed in triplicates for at least three independent assays. Cell viability was monitored by MTS measurement using the CellTiter 96 Aqueous One solution (Promega) according to the manufacturer’s recommendations.

### Real Time PCR

HT-29 cell line was seeded in 6-well culture plates at densities of 10^6^ cells per well and cultured for 24h prior stimulation. Total messenger RNA (mRNA) was extracted after 6h incubation with CDM, CDM and rosiglitazone (10μM), or supernatant and rosiglitazone using an RNeasy mini-kit (Qiagen), cDNA was synthesized from 2 μg of mRNA using High Capacity cDNA Reverse Transcription Hits (Applied biosystems) according to the manufacturer’s instructions. qPCRs were carried out using thermal cycler in a reaction volume of 25μl and SYBR Green (Applied Biosystems)-based quantitative real-time PCR. Primers were designed and tested according to Applied Biosystems recommendations. Liver-Fatty acid binding protein (L-FABP), 5’-GGAAGCACTTCAAGTTCACCAT-3’/5’-ACCTTCCAACTGAACCACTGTC-3’; β-actin (used for normalization), 5’-AAGACCTGTACGCCAACACAGT-3’/5’-GGAGCAATGATCTTGATCTTCA-3’; Angiopoietin like protein-4 (*Angptl4*), 5’-AGGCTGGACAGTAATTCAGAGG-3’/5’-ATGCTATGCACCTTCTCCAGAC-3’; Peroxisome Proliferator-Activated Receptor-γ (PPARγ), 5’-TCCAGTGGTTGCAGATTACAAG3’/5’-AGGCTCTTCATGAGGCTTATTG-3’. The sample setups included biological duplicates and experimental triplicates.

### Western-blot

Protein extracts were run in 10% SDS-PAGE and transferred onto PVDF membranes (Bio-Rad, Transblot). Membranes were blocked overnight in PBS/4% skim milk/0.1% Tween-20 (Sigma-aldrich). Primary (anti-PPARγ, SC-7273, clone E-8; anti-GAPDH, SC-365062, clone G-9; both from Santa Cruz Biotechnology) and secondary (Goat anti-mousse IgG HRP, Dako, P0447) antibodies were successively added in PBS/Tween/milk, each being left for 1h before detection with the Clarity Western ECL Substrate using the Chemidoc MP System (Bio-Rad). Quantifications were performed using the image Lab software (Bio-Rad).

### Statistical analysis

Graphic representation and statistical analyses of PPARγ, *Angptl4* and FABP expressions by RT-qPCR were performed using Graphpad Prism software. Comparisons of distributions were performed using a student’s t test with 95% confidence intervals with p values of ≤0.05 considered to be significant.

## Results

### *S*. *salivarius* strains inhibit PPARγ activity

We previously demonstrated that *S*. *salivarius* strains inhibit NF-κB activation in IECs through an unknown mechanism [34]. PPARγ is a well-characterized nuclear receptor recently reported to act as an E3 ubiquitin ligase implicated in the degradation of the p65 sub-unit of NF-κB and consequently reducing inflammatory responses [19]. As NF-κB and PPARγ are transcription factors targeted by bacteria including commensals [20, 34], we aimed to understand if *S*. *salivarius*-dependent down-modulation of NF-κB was linked to the induction of PPARγ transcriptional activity.

**Fig 1:**
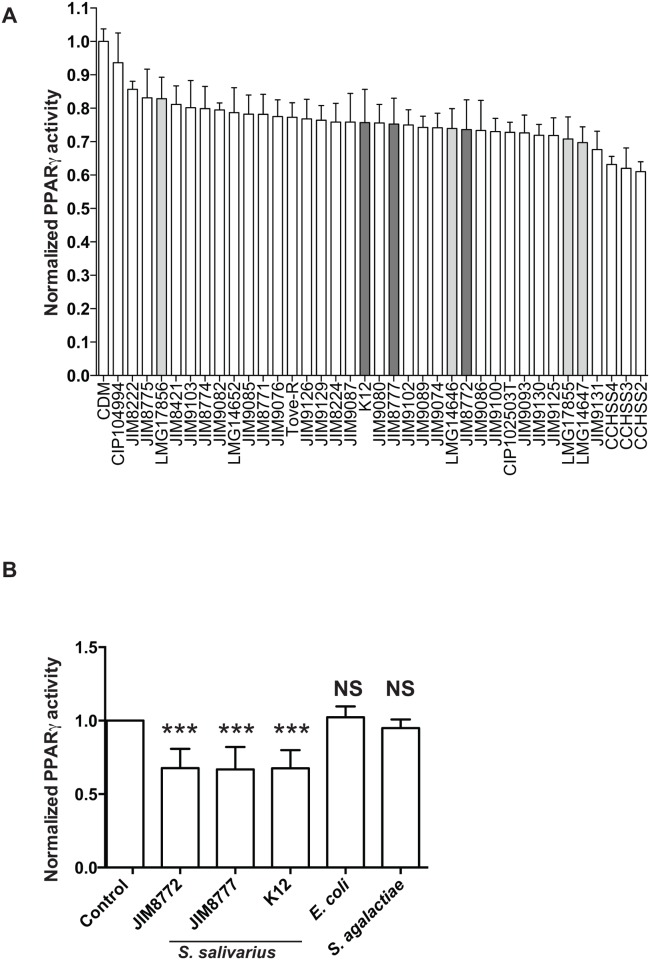
*S*. *salivarius* and *S*. *vestibularis*, but not *S*. *agalactiae* and *E*. *coli*, culture supernatants down-regulate butyrate-induced PPARγ activity in HT-29/PPARγ. A- Culture supernatants of a wide range of *S*. *salivarius* (white bars) and *S*. *vestibularis* (grey bars) strains were applied on HT-29-PPARγ (10% vol/vol) prior to PPARγ-induction by sodium butyrate (But, 2mM) for 12h. PPARγ transcriptional activity was measured by luciferase activity and expressed as fold increase towards its control: growth-medium + butyrate. Data are expressed as means ± standard deviations (SD) of triplicate measurements from one representative experiment out of three independent experiments. The dark grey bars represent the selected *S*. *salivarius* strains used for the remaining of the study (JIM8772, JIM8777 and K12). B- Culture supernatants of three *S*. *salivarius* (JIM8772, JIM8777 and K12), *E*. *coli* and *S*. *agalactiae* strains were applied on HT-29-PPARγ (10% vol/vol) prior to PPARγ-induction by sodium butyrate (2mM) for 12h. PPARγ expression was measured by luciferase activity and expressed as fold increase towards growth-medium + sodium butyrate (Control). Data are expressed as means ± standard deviations (SD) of triplicate measurements from one representative experiment out of three independent experiments. ***P<0.001 compared with controls (Student's t-test).

For this purpose, we used a PPARγ reporter system under transcriptional control by three repeats of a PPRE (PPARγ-responsive element) stably expressed in HT-29 intestinal epithelial cells [12]. We screened the effect of bacterial supernatants derived from a wide range of *S*. *salivarius* and *vestibularis* strains isolated from diverse human sites previously described to modulate NF-κB pathway on the PPARγ reporter system [34]. Using non-activated cells, none of the strains tested showed any effect on PPARγ activation *per se*, suggesting that PPARγ is not involved in *S*. *salivarius*-dependent NF-κB inhibition (S1 Fig). Interestingly, upon activation of PPARγ by butyrate supernatants secreted by *S*. *salivarius* and *S*. *vestibularis* strains down-regulated activity with an inhibition rate, ranging from 15% to 40%. (Fig 1A). Indirect effects through cell viability were excluded using the MTS assay (S2 Fig). This repression was specific to *S*. *salivarius* and *S*. *vestibularis* supernatants as *E*. *coli* and *S*. *agalactiae* supernatants did not affect butyrate-induced PPARγ transcriptional activity (Fig 1B).

### The *S*. *salivarius*–dependent down-regulation of PPARγ transcriptional activity relies on secreted molecules

**Fig 2:**
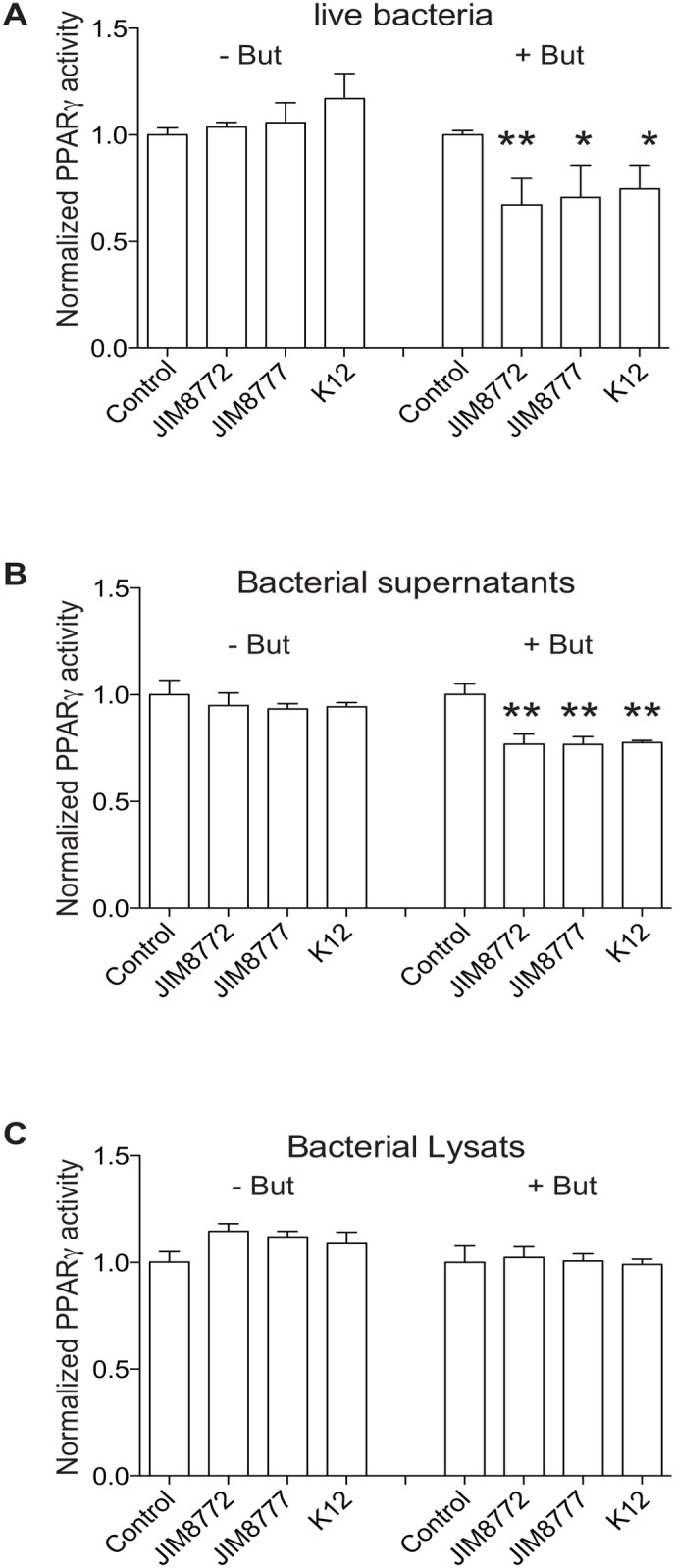
The *S*. *salivarius*–dependent down-regulation of PPARγ activity relies on secreted molecules. PPARγ was activated (+ But) or not (- But) in HT-29 reporter cells using sodium butyrate (2mM) in the presence of control medium (Control) or as indicated live bacteria (A), bacterial supernatants (B) or lysates (C). The values represent the luciferase activity normalized towards their respective control. Data are expressed as means ± standard deviations (SD) of triplicate measurements from one representative experiment out of three independent experiments. ***P<0.001, **P<0.005, compared with controls (Student's t-test).

Growth media are highly enriched in molecules, which are potentially metabolized by bacteria. In order to avoid growth medium-derived effects in PPARγ down-regulation, we used living bacteria from exponentially growing cultures that were washed with PBS prior to application to the PPARγ-HT-29 reporter cell line. In Fig 2, we showed that live *S*. *salivarius* strains, similarly to their culture supernatants inhibited the activation of butyrate-induced PPARγ on intestinal epithelial cells (Fig 2A and 2B). Moreover, bacterial cell components are known to be released during replication and lysis in exponentially growing cultures. In order to test if the observed PPARγ down-regulation with bacterial supernatants resulted from cytoplasmic or membranous compounds that might have been released during growth, we tested lysates of the washed bacteria on HT-29-PPARγ reporter cell-line but did not find any significant changes in PPARγ activation (Fig 2C).

These results strongly suggest an effect on butyrate-induced PPARγ activation exclusively with secreted components from metabolically active bacteria.

### The *S*. *salivarius*–dependent down-regulation of PPARγ activity is not dependent of the epithelial cell-line or PPARγ ligands used

**Fig 3:**
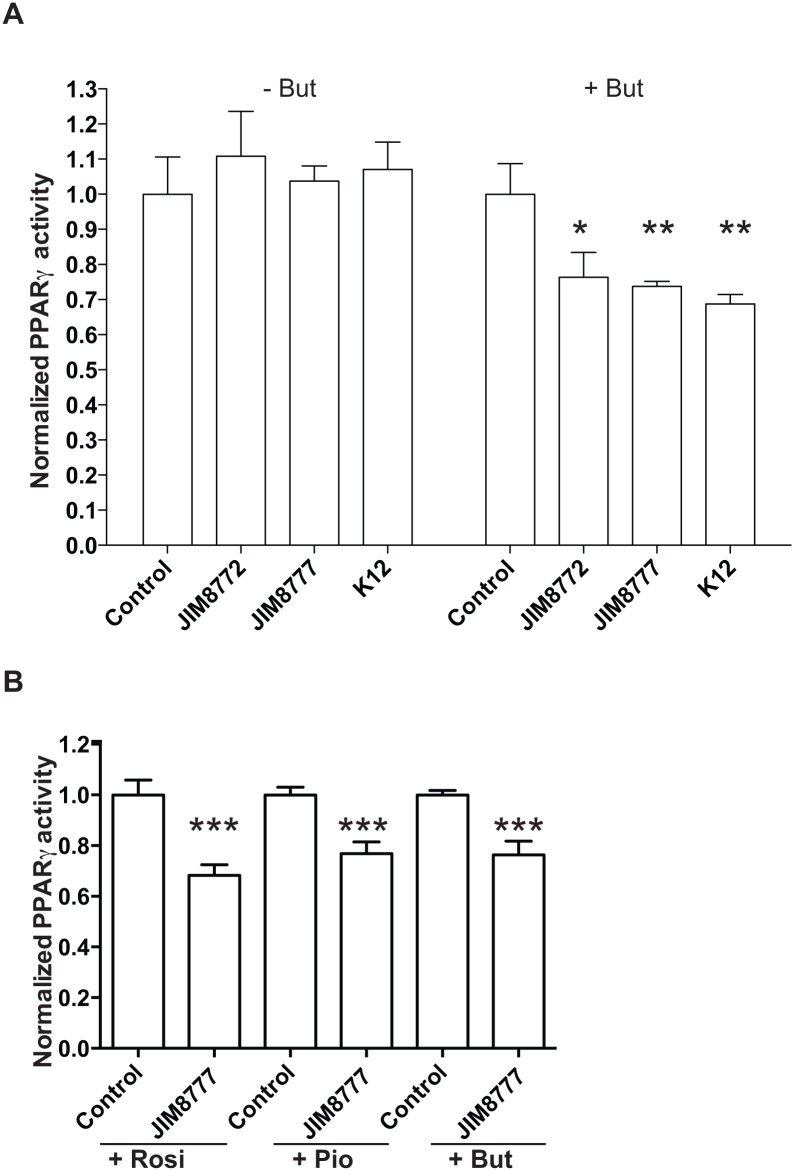
The *S*. *salivarius*–dependent down-regulation of PPARγ activity is independent of the epithelial cell-line or the specific PPARγ-ligand used. A- PPARγ was activated (+ But) or not (- But) in Caco-2 reporter cells (Caco2-PPARγ) using sodium butyrate (2mM) in the presence of control medium (Control) or supernatants. B- HT-29-PPARγ reporter cell-line was activated with different activators: rosiglitazone (Rosi, 10μM), pioglitazone (Pio, 10μM) and sodium butyrate (But, 2mM) in presence of *S*. *salivarius* JIM8777 supernatant. The values represent the luciferase activity normalized towards their respective control. Data are expressed as means ± standard deviations (SD) of triplicate measurements from one representative experiment out of three independent experiments. ***P<0.001, **P<0.005, *P<0,05 compared with controls (Student's t-test).

*S*. *salivarius* effects on PPARγ activation was also confirmed in a second epithelial cell line, i.e. Caco-2, carrying the same reporter system (Fig 3A). Supernatants from three *S*. *salivarius* strains down-regulated butyrate-induced PPARγ activation in Caco-2 cells in a similar range as observed in HT-29 (30 to 40%).

Butyrate is a short chain fatty acid (SCFA) with broad cellular activities through receptor activation such as GPR41 and GPR43 or histone deacetylates (HDAC) inhibition [12]. The observed down-regulation of butyrate mediated PPARγ activation by *S*. *salivarius* and *S*. *vestibularis* supernatants could therefore be mediated either through direct PPARγ inhibition or indirectly through inhibition of the activation mechanisms of butyrate. We therefore repeated our experiments with a range of well-characterized specific PPARγ ligands inducing its translocation to the nucleus and consequently enhancing its transcriptional activity (Fig 3B and S3 Fig). Interestingly, bacterial supernatants inhibited PPARγ activation independently of the PPARγ ligand used (rosiglitazone, pioglitazone, sodium butyrate) suggesting a ligand-aspecific effect and potentially a common downstream regulation of PPARγ activation pathway (Fig 3B).

### The secreted bioactive component is a small, non nucleotidic, non peptidic, heat-resistant molecule

**Fig 4:**
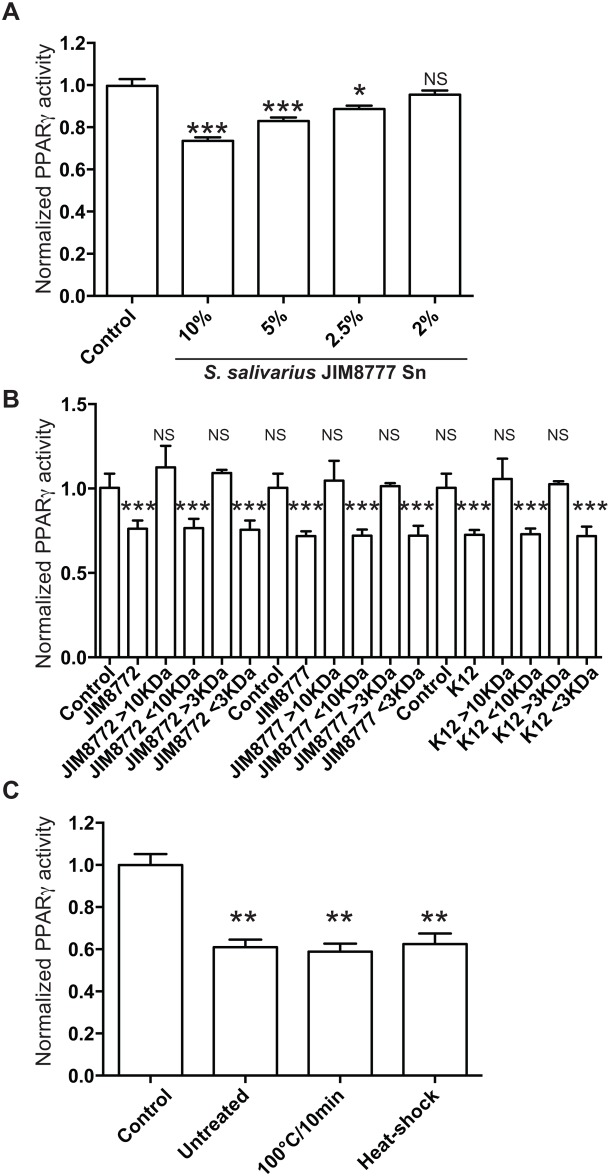
Determination of the nature and molecular mass of the secreted bioactive compounds. A- Serial dilutions of *S*. *salivarius* JIM8777 supernatant were tested on HT-29-PPARγ cells prior to activation with sodium butyrate (2mM). B- Exposure to high temperature (100°C/10 min) and heat shock were applied to *S*. *salivarius* JIM8777 supernatant prior to addition to activated HT-29-PPARγ cells. C- Butyrate-activated HT-29-PPARγ cells were incubated with *S*. *salivarius* supernatants fractions derived from ultrafiltration through 3 and 10 kDa cutoff membranes. >10kDa/>3kDa and <10kDa/<3kDa represent the retained and filtered fractions respectively. Data are expressed as means ± standard deviations (SD) of triplicate measurements from one representative experiment out of three independent experiments. ***P<0.001, **P<0.005, *P<0,05 compared with controls (Student's t-test).

**Fig 5:**
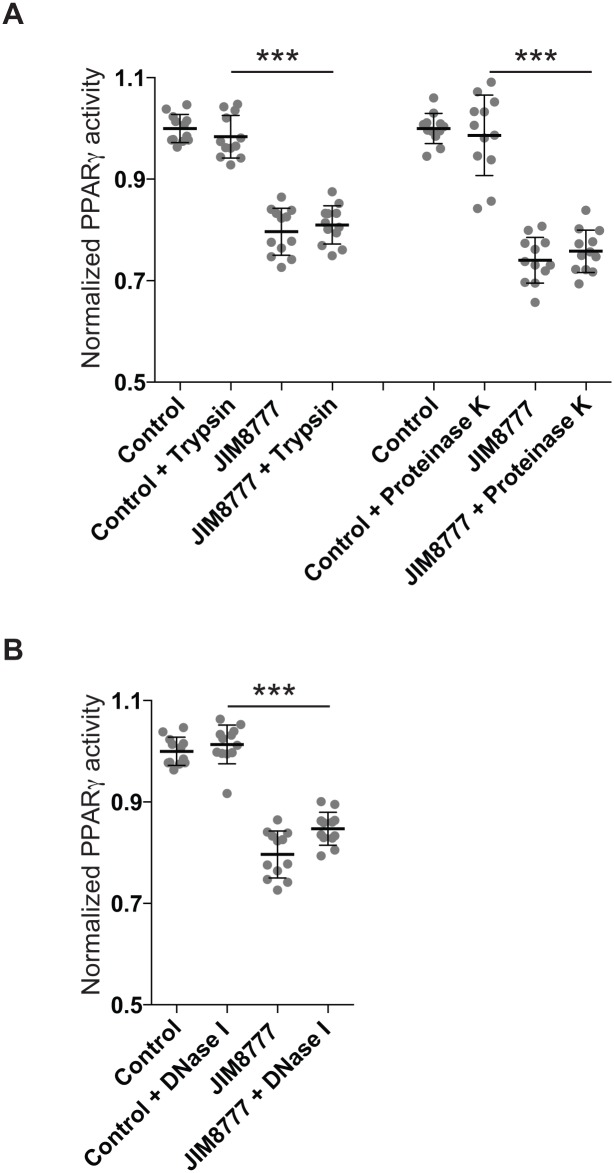
The *S*. *salivarius* bioactive compounds are not from peptidic or nucleotidic origin. A- 3kDa-filtered *S*. *salivarius* JIM8777 supernatants were exposed to proteases (trypsin, left panel; proteinase K, right panel) prior to addition to butyrate-activated HT-29-PPARγ cells. B- 3kDa-filtered *S*. *salivarius* JIM8777 supernatants were exposed to DNase I prior to addition to butyrate-activated HT-29-PPARγ cells.

By serial dilution of the bacterial supernatants, we showed that the observed inhibitory effect was dose-dependent and detected at a concentration of 2.5% of supernatant in the culture media suggesting that the bioactive compound was expressed in a significant amount (Fig 4A). Standard size-cutoff columns were used to assess the size of the active(s) compound(s) secreted by *S*. *salivarius*. Bacterial supernatants were submitted to ultrafiltration through 3 and 10 kDa cutoff membranes. For the three strains tested, the <10 kDa and <3 kDa fractions inhibited PPARγ activity similarly to the unfiltered fractions while fractions > 10kDa and >3 kDa displayed no effect suggesting that the active compound was a small molecule lower than 3kDa (Fig 4B). Moreover, exposure to high temperatures (100°C for 10 min) or to heat shock (100°C for 10 min before freezing in liquid nitrogen) did not affect the inhibitory property of the supernatant (Fig 4C). Furthermore, the treatments of *S*. *salivarius* supernatants with proteases (proteinase K and trypsin) or DNase I did not impair the inhibition of butyrate-induced PPARγ activity (Fig 5). All together, these results showed that the bioactive compound is a small non-peptidic and non-nucleotidic heat-resistant molecule.

### *S*. *salivarius* modulates PPARγ-dependent gene expression, I-FABP and *Angptl4*,without affecting PPARγ expression

**Fig 6:**
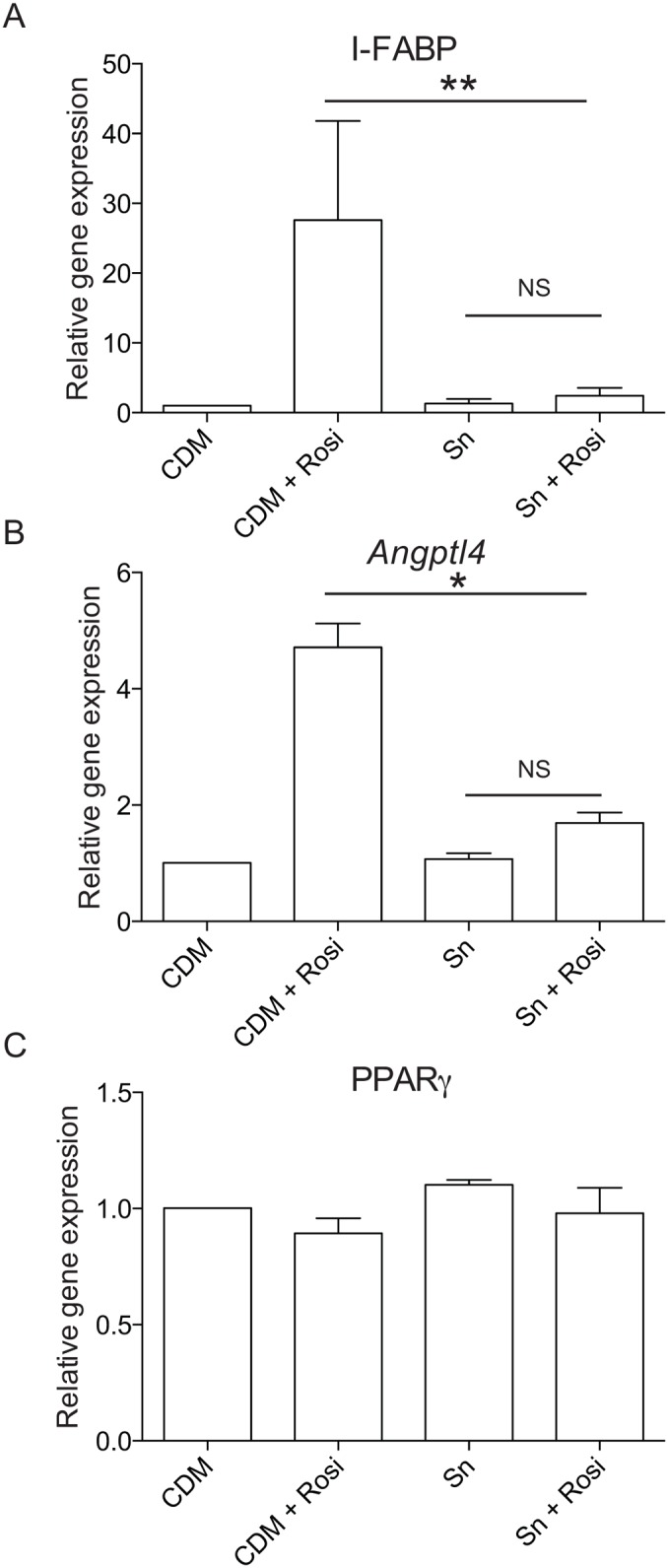
Transcriptional regulation of PPARγ and PPARγ-target genes upon stimulation with rosiglitazone and/or *S*. *salivarius* supernatant. The mRNA expression of *I-FABP* (A), *Angptl4* (B) and *PPARγ* (C) were determined by Quantitative real-time PCR on total RNA extracted from HT-29 cells exposed to culture medium (CDM), *S*. *salivarius* JIM8777 supernatant (Sn) alone or in addition with the PPARγ specific activator rosiglitazone (CDM+Rosi; Sn+Rosi) for 6 h. Expression is represented as fold change compared to the absence of any stimulation (CDM medium only). Data are represented as mean ± standard deviation (SD) of 2 to 3 independent repetitions done in triplicates. **P<0.005, *P<0,05 compared with controls (Student's t-test).

**Fig 7:**
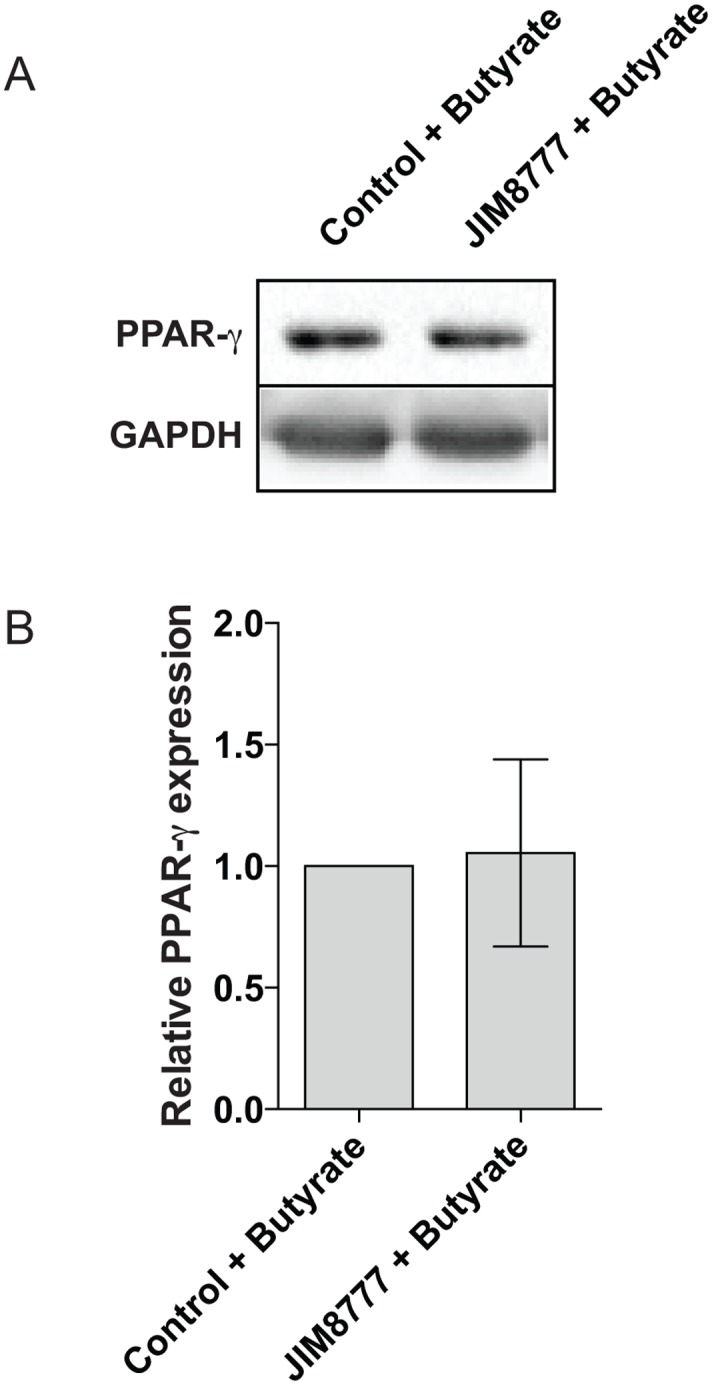
PPARγ protein level is unchanged upon stimulation with sodium butyrate and/or *S*. *salivarius* supernatant. A- The protein level of PPARγ and GAPDH were determined by western-blot on total protein extracted from HT-29 cells exposed to culture medium (CDM), *S*. *salivarius* JIM8777 supernatant in addition with sodium butyrate (Control + Butyrate; JIM8777 + Butyrate) for 24 h. B- Quantifications of total PPARγ protein normalized to GAPDH protein level. Protein expression is represented as fold change compared to the sodium butyrate stimulation in presence of culture media (Control + Butyrate). Data are represented as mean ± standard deviation (SD) of the effect of 5 independent bacterial cultures.

**Fig 8:**
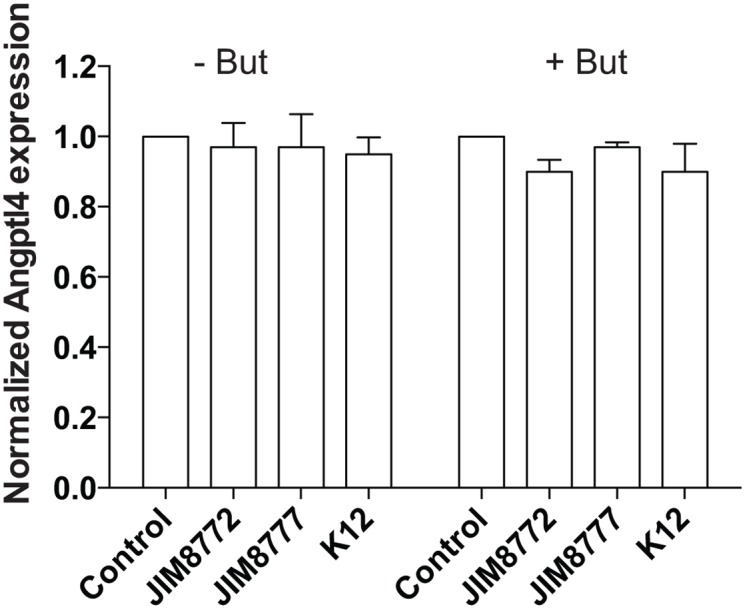
*S*. *salivarius* supernatants do not affect PPARγ-independent ANGPTL4 reporter system. ANGPTL4-reporter system cell-line was activated with sodium butyrate (2mM) with or without *S*. *salivarius* supernatant (JIM8772, JIM8777 or K12). *Angtpl4* expression was measured by luciferase activity and expressed as fold increase towards its control: growth-medium alone (left panel) and growth-medium + sodium butyrate (right panel). Data are expressed as means ± standard deviations (SD) of triplicate measurements from one representative experiment out of three independent experiments. ***P<0.001, **P<0.005, *P<0,05 compared with controls (Student's t-test).

To assess the impact of *S*. *salivarius*-dependent inhibition on PPARγ target genes, we quantified by RT-qPCR the expression of *Angptl4* and *I-FABP* (intestinal fatty acid binding protein). The expression of these two well-characterized PPARγ-regulated genes were strongly reduced by *S*. *salivarius* supernatant in the presence of the PPARγ-specific ligand, rosiglitazone (Fig 6A and 6B). This inhibitory effect was independent on *PPARγ* expression as its expression level was unchanged when HT-29 cells were incubated with both supernatant and the specific ligand rosiglitazone (Fig 6C). Moreover, *S*. *salivarius* did not decrease the stability of PPARγ protein as it remained identical after bacterial supernatant treatment (Fig 7). To confirm these observations, we used a cell-line expressing a ANGPTL4 reporter system containing the 1.8 kb of ANGPTL4 promoter devoid of the functional PPRE present in the third intron of the gene [12]. As shown in Fig 8, none of the *S*. *salivarius* supernatants tested were able to affect the PPARγ-independent *Angptl4* expression induced by sodium butyrate.

Together, these findings show that S. salivarius supernatants specifically repressed the PPARγ pathway without affecting PPARγ gene expression and protein stability, and consequently can impact metabolic responses.

## Discussion

Commensal bacteria provide crucial biological functions to their host, including metabolic functions and the control of moderate immunological responses, beneficial for human health. This intimate relationship results from a balanced interaction between the host and its microbiota mediated through direct contact or secreted active compounds. The colonization of the human digestive tract by commensals occurs immediately after birth and has been shown by a growing number of evidence to be the key factor directly impacting host physiology [42–45]. However, while most studies concern adult or predominant gut microbiota, very little is known about the impact of early bacterial colonizers on human physiology. The commensal bacterium *Streptococcus salivarius* is one of the primo-colonizers of oral and gut mucosal surfaces that have been shown to influence inflammation by down-modulating NF-κB activity in human intestinal cells [33, 34]. The present study focused on the direct impact of *S*. *salivarius* on a key transcription factor in intestinal epithelial cells (IEC) that links metabolism and inflammation, namely PPARγ.

One of PPARγ’s immunomodulatory properties is to inhibit NF-κB activation by directly impairing its translocation into the nucleus or by acting as an E3 ligase targeting the p65 sub-unit of NF-κB for degradation [19]. We previously showed that *S*. *salivarius* strains inhibit NF-κB activation in IECs via unknown pathways leading to *in vivo* protection in induced-colitis mice models [33, 34]. Since several studies have linked NF-κB control and PPARγ activity, it was thus conceivable that *S*. *salivarius*-dependent down-modulation of NF-κB could be linked to the induction of PPARγ transcriptional activity [19, 20]. Using human intestinal epithelial cell-lines engineered to monitor PPARγ transcriptional activity in response to a wide range of *S*. *salivarius* strains, we demonstrate that different members of the *S*. *salivarius* group share the capacity to inhibit PPARγ transcriptional activity. This result suggests that PPARγ is not involved in the NF-κB-dependent anti-inflammatory properties of *S*. *salivarius*. Moreover, these results together with our previous study suggest that species belonging to the streptococcal salivarius group of commensal streptococci are presenting effects both on host inflammatory regulation and on metabolism processes [34].

First attempts to decipher the nature of the active compound showed that it is a low-molecular-weight metabolite (<3kDa) produced and secreted by *S*. *salivarius*. Moreover, exposure to enzymatic and heat treatments did not affect the inhibitory potential suggesting that it may be a small organic molecule of non-peptidic and non-nucleotidic origin. The phylogenetic proximity of the strains tested suggests a potential related metabolite production pathway inherited from a common ancestor [46]. We and others showed that common bacterial metabolites such as short-chain fatty acids (SCFAs) regulate PPARγ activity [12, 41]. It is unlikely that SCFAs are involved in PPARγ down-modulation as acetate, butyrate and propionate have an opposite effect to *S*. *salivarius* supernatant by inducing the up-regulation of PPARγ responses (S3 Fig and [41]). Moreover S. *salivarius* is not reported to produce butyrate, propionate and acetate at activating concentrations [34]. We also ruled out that the inhibitory effect was due to lactic acid known to be produced by *S*. *salivarius*. Indeed, high-pressure liquid chromatography (HPLC) measurement of organics revealed a maximal concentration of lactic acid around 80 mM in *S*. *salivarius* supernatant [34]. We assess the effect of lactic acid on our reporter systems and observe no effect on PPARγ activity (data not shown). Interestingly, on the contrary to what was observed for NF-κB down-modulation, *S*. *salivarius* derived compounds inhibiting PPARγ are resistant to trypsin treatment ruling out a component with a pleiotropic activity on the host.

Recently, pathogen and commensal bacteria have been shown to actively modulate PPARγ transcriptional activity mainly *via* up-regulating its expression (including *Helicobacter pylori*, *Bacteroidetes thetaiotaomicron*, *Brucella abortus*, *Salmonella Typhimurium*, *Mycobacterium tuberculosis*, *Neisseria lactamica* and short chain fatty acids derived from commensals) [12, 13, 20, 35, 37, 47–49]. Alternatively, PPARγ activity is induced by phosphorylation via unknown bacterial molecules derived from *Enterococcus faecalis* or by direct interaction with lipids derived from *M*. *tuberculosis* [13, 48]. For pathogens, a direct correlation between PPARγ up-regulation and bacterial survival has been observed [48]. On the contrary to what is observed with these pathogens, we showed that PPARγ activity is impaired by *S*. *salivarius* supernatants. So far, only *Salmonella typhimurium* has been shown to inhibit PPARγ activity in mouse epithelial cells by down-regulating its gene expression, aggravating acute colitis [35]. However, common mechanisms are unlikely to occur as we showed by RT-qPCR and western-blotting that *PPARγ* gene and protein expression are unchanged in presence of *S*. *salivarius*-derived supernatant (Figs 6C and 7).

Endogenous ligands of PPARγ such as polyunsaturated fatty acids and their derivatives, along with the synthetic anti-diabetic drugs derived from thiazolidinediones such as rosiglitazone and pioglitazone promote heterodimers formation with the retinoid X receptor (RXR), and subsequent recognition of PPAR-response elements (PPREs) within target gene promoters. Additionally to ligand-dependent activation, PPARγ has been shown to be phosphorylated, SUMOylated and ubiquitinated regulating its transcriptional properties (for review see [50]). These diverse regulatory processes regulating PPARγ activity offer numerous possible mechanisms by which PPARγ transcriptional activity might be inhibited by *S*. *salivarius* supernatants. Our present study shows that *S*. *salivarius* supernatant inhibits PPARγ transcriptional activity induced in epithelial cells even by highly specific ligands such as rosiglitazone, pioglitazone or butyrate, a SCFA. Absence of repression on the basal PPARγ activity suggests a putative competition in the activation of the PPARγ pathway (Fig 1A). Butyrate-induced signaling cascade involve an increased expression of PPARγ mRNA whereas synthetic ligands induce its dimerization and phosphorylation suggesting that a common regulation in the early pathway is unlikely. Although the exact mechanisms remain unclear, the fact that *S*. *salivarius* supernatant inhibits PPARγ independently of the activator used, along with the nature of the inhibitory molecules suggest its possible SUMOylation known to recruit a co-repressor complex impairing PPARγ translocation or the implication of a ligand or regulatory pathways with direct antagonist effects [50].

Considering the multitude of strategies evolved in pathogenic bacteria to modulate the host environment in order to multiply and spread, it is not surprising that commensal microorganisms have co-evolved similarly to survive in highly competitive niches such as mucosal sites. *H*. *pylori* and *M*. *tuberculosis* up-regulate host PPARγ expression to suppress exaggerated inflammatory responses, ensuring their survival within the host [48, 49]. In contrast, *S*. *typhimurium* initiates an acute inflammation by inducing PPARγ down-regulation in the intestinal epithelium, leading to the development of a hostile niche for local competitors that will favor its survival [35]. It is therefore tempting to speculate that PPARγ-inhibition induced by *S*. *salivarius* is a mechanism to thwart pathogen’s strategy or to enhance its own survival. However infection or colonization trials on animal models using *S*. *salivarius* as a PPARγ inhibitor would be needed to prove this hypothesis. Studies in animals have demonstrated that *S*. *salivarius* significantly inhibited inflammation in TNBS-induced colitis mouse models suggesting that the PPARγ-independent inhibition of NF-κB counterbalances the inhibition of PPARγ transcriptional activity observed in our study [33].

Despite being a master regulator of inflammation, by antagonizing the activities of the transcription factors AP-1, STAT, and NF-κB and inducing visceral adipose tissue (VAT)-resident regulatory T cells (Treg), PPARγ has a broad range of physiological properties including the regulation of lipid and glucose metabolism [20, 51–53]. Among PPARγ-targeted metabolic genes, *I-FABP* and *Angptl4* levels are dramatically reduced in presence of *S*. *salivarius*. Both of these gene products modulate lipid accumulation in cells and down-regulating their expression might consequently be detrimental for host health [18, 54–56]. However, the expression of lipid metabolic factors might be important in the weaning or early post-weaning to deal with a highly rich nutritional intake when *S*. *salivarius* is particularly dominant. It is therefore tempting to speculate that intestinal PPARγ may be one of several transcription factors controlling genes involved in lipid metabolism such as *Angptl4* under the influence of microbiota and recently reported in studies of obese patients [18, 56, 57]. We believe that a combination of commensal bacteria can be involved in transcriptional regulation through factors such as PPARγ, and that the consequence of such regulations are a tightly tuned balance of immune and metabolic genes. Studies of *S*. *salivarius* in high-fat diet obese or post-weaning mice models would help to decipher this important issue.

Here we demonstrate that strains of *S*. *salivarius*, an early colonizer of the gastrointestinal tract, can inhibit the activity of the transcription factor PPARγ and the subsequent expression of target genes in intestinal epithelial cell lines. By showing that a commensal bacterium targets such type of nuclear receptor with wide activity, this study reinforces the notion that the microbiota contributes to immune and metabolic regulation that are highly interconnected in intestinal cells.

## Supporting Information

S1 Fig *S*. *salivarius* and *S*. *vestibularis* culture supernatants do not impair steady-state PPARγ activity in HT-29/PPARγ. Culture supernatants of a wide range of *S*. *salivarius* (white bars) and *S*. *vestibularis* (grey bars) strains were applied on HT-29-PPARγ (10% vol/vol) without any PPARγ activation for 12h. PPARγ transcriptional activity was measured by luciferase activity and expressed as fold increase towards its control: growth-medium alone. Data are expressed as means ± standard deviations (SD) of triplicate measurements from one representative experiment out of three independent experiments. The dark grey bars represent the selected *S*. *salivarius* strains used for the remaining of the study (JIM8772, JIM8777 and K12).
(EPS)

S2 Fig *S*. *salivarius* supernatants do not affect cell viability of butyrate-induced HT-29 cells. Cell viability was monitored on HT-29 cells by MTS measurement after incubation for 12h with butyrate alone (Control), sodium butyrate + growth media (CDM) or sodium butyrate + culture supernatant from *S*. *salivarius* (JIM8772, JIM8777 or K12). Data are expressed as means ± standard deviations (SD) of triplicate measurements from one representative experiment out of three independent experiments.
(EPS)

S3 Fig HT-29-PPARγ cell-line responses to different PPARγ ligands (rosiglitazone, pioglitazone, tioglitazone, ciglitazone, 10μM). The values represent the luciferase activity normalized towards the negative control (untreated cells). Data are expressed as means ± standard deviations (SD) of triplicate measurements from one representative experiment out of three independent experiments.
(EPS)

S1 Table Bacterial strains used in this study. (DOCX)
