# Supplementary material for: Commensal Streptococcus salivarius Modulates PPARγ Transcriptional Activity in Human Intestinal Epithelial Cells
Source: PLoS One. 2015 May 6;10(5):e0125371. doi: 10.1371/journal.pone.0125371 (PMC4422599; doi:10.1371/journal.pone.0125371)
Supplement: S1 Table — (DOCX) [file pone.0125371.s004.docx]

**S1 Table:** **Bacterial strains used in this study.**

| Species | Strains | Reference | Source |
| --- | --- | --- | --- |
| *S. salivarius* | CCHSS2 | [[1](#_ENREF_1)] | Human blood |
|  | CCHSS3 | [[2](#_ENREF_2)] | Human blood |
|  | CCHSS4 | [[1](#_ENREF_1)] | Human blood |
|  | CIP102503T | [[1](#_ENREF_1)] | Human blood |
|  | CIP104994 | [[1](#_ENREF_1)] | Human blood |
|  | JIM8222 | [[1](#_ENREF_1)] | Oral cavity |
|  | JIM8224 | [[1](#_ENREF_1)] | Oral cavity |
|  | JIM8421 | [[1](#_ENREF_1)] | Breast milk |
|  | JIM8771 | [[1](#_ENREF_1)] | Oral cavity |
|  | JIM8772 | [[1](#_ENREF_1)] | Oral cavity |
|  | JIM8774 | [[1](#_ENREF_1)] | Oral cavity |
|  | JIM8775 | [[1](#_ENREF_1)] | Oral cavity |
|  | JIM8777 | [[3](#_ENREF_3)] | Oral cavity |
|  | JIM9074 | This work | Human blood |
|  | JIM9076 | This work | Sputum |
|  | JIM9080 | This work | Peritoneal cavity |
|  | JIM9082 | This work | Peritoneal cavity |
|  | JIM9085 | This work | Peritoneal cavity |
|  | JIM9086 | This work | Peritoneal cavity |
|  | JIM9087 | This work | Human blood |
|  | JIM9089 | This work | Human blood |
|  | JIM9093 | This work | Human blood |
|  | JIM9100 | This work | Human blood |
|  | JIM9102 | This work | Peritoneal cavity |
|  | JIM9103 | This work | Peritoneal cavity |
|  | JIM9125 | [[4](#_ENREF_4)] | Oral cavity |
|  | JIM9126 | [[4](#_ENREF_4)] | Oral cavity |
|  | JIM9129 | This work | Oral cavity |
|  | JIM9130 | This work | Oral cavity |
|  | JIM9131 | This work | Oral cavity |
|  | K12 | [[5](#_ENREF_5)] | Saliva from healthy child |
|  | LMG14652 | [[1](#_ENREF_1)] | Human blood |
|  | Tove-R | [[6](#_ENREF_6)] | Oral cavity |
| *S. vestibularis* | LMG14646 | [[7](#_ENREF_7)] | Vestibular mucosa |
|  | LMG14647 | [[7](#_ENREF_7)] | Vestibular mucosa |
|  | LMG17855 | [[7](#_ENREF_7)] | Vestibular mucosa |
|  | LMG17856 | [[7](#_ENREF_7)] | Human dental plaque |
| *S. agalactiae* | NEM316 | [[8](#_ENREF_8)] | Neonate human blood |
| *E. coli* | EPI300 | Epicentre |  |

# References

1. Delorme C, Poyart C, Ehrlich SD, Renault P. Extent of horizontal gene transfer in evolution of Streptococci of the salivarius group. Journal of bacteriology. 2007;189(4):1330-41. doi: 10.1128/JB.01058-06. PubMed PMID: 17085557; PubMed Central PMCID: PMC1797340.

2. Delorme C, Guedon E, Pons N, Cruaud C, Couloux A, Loux V, et al. Complete genome sequence of the clinical Streptococcus salivarius strain CCHSS3. Journal of bacteriology. 2011;193(18):5041-2. doi: 10.1128/JB.05416-11. PubMed PMID: 21742894; PubMed Central PMCID: PMC3165645.

3. Guedon E, Delorme C, Pons N, Cruaud C, Loux V, Couloux A, et al. Complete genome sequence of the commensal Streptococcus salivarius strain JIM8777. Journal of bacteriology. 2011;193(18):5024-5. doi: 10.1128/JB.05390-11. PubMed PMID: 21742871; PubMed Central PMCID: PMC3165664.

4. Kaci G, Lakhdari O, Dore J, Ehrlich SD, Renault P, Blottiere HM, et al. Inhibition of the NF-kappaB pathway in human intestinal epithelial cells by commensal Streptococcus salivarius. Applied and environmental microbiology. 2011;77(13):4681-4. doi: 10.1128/AEM.03021-10. PubMed PMID: 21602373; PubMed Central PMCID: PMC3127691.

5. Barretto C, Alvarez-Martin P, Foata F, Renault P, Berger B. Genome sequence of the lantibiotic bacteriocin producer Streptococcus salivarius strain K12. Journal of bacteriology. 2012;194(21):5959-60. doi: 10.1128/JB.01268-12. PubMed PMID: 23045482; PubMed Central PMCID: PMC3486084.

6. Tanzer JM, Kurasz AB, Clive J. Competitive displacement of mutans streptococci and inhibition of tooth decay by Streptococcus salivarius TOVE-R. Infection and immunity. 1985;48(1):44-50. PubMed PMID: 3980093; PubMed Central PMCID: PMC261912.

7. Delorme C, Bartholini C, Bolotine A, Ehrlich SD, Renault P. Emergence of a cell wall protease in the Streptococcus thermophilus population. Applied and environmental microbiology. 2010;76(2):451-60. doi: 10.1128/AEM.01018-09. PubMed PMID: 19915034; PubMed Central PMCID: PMC2805209.

8. Glaser P, Rusniok C, Buchrieser C, Chevalier F, Frangeul L, Msadek T, et al. Genome sequence of Streptococcus agalactiae, a pathogen causing invasive neonatal disease. Molecular microbiology. 2002;45(6):1499-513. PubMed PMID: 12354221.
